# Outcome of Surgical Treatment of Giant Cell Tumors of Bone Around the Knee Joint for Extended Curettage or Segmental Resection: A Retrospective Study

**DOI:** 10.7759/cureus.86766

**Published:** 2025-06-25

**Authors:** Nishant Kashyap, Abhijeet Subhash, Wasim Ahmed, Ritesh Runu, Santosh Kumar, Indrajeet Kumar

**Affiliations:** 1 Orthopaedics, Indira Gandhi Institute of Medical Sciences, Patna, Patna, IND

**Keywords:** extended curettage, giant cell tumor, msts score, recurrence, segmental resection

## Abstract

Background

Giant cell tumors (GCTs) of bone around the knee are locally aggressive benign neoplasms with a tendency for recurrence and functional compromise. Surgical options include extended curettage (EC), often combined with adjuvants and internal fixation (sandwich technique), or segmental resection (SR) with megaprosthesis reconstruction. The optimal approach remains debated. This study compares oncological and functional outcomes between these two surgical strategies in a cohort of 65 patients.

Methods

A retrospective analysis was conducted at the Department of Orthopaedics, Indira Gandhi Institute of Medical Sciences, Patna, over six years. Patients with biopsy-confirmed GCTs around the knee who underwent EC or SR were included. Outcomes assessed included local recurrence, recurrence-free survival (RFS), Musculoskeletal Tumor Society (MSTS) scores, operative time, blood loss, hospital stay, and postoperative complications.

Results

Of the 65 patients (EC: 48; SR: 17), local recurrence was noted in 12.5% of the EC group and 5.9% of the SR group (p=0.762). For Grade II tumors, recurrence occurred in 4.2% of EC cases and none in SR; for Grade III tumors, recurrence rates were 8.3% (EC) and 5.9% (SR). At three years, RFS was 87.5% for EC and 94.1% for SR (p=0.604). SR involved longer surgeries (172.7 ± 36.3 vs. 119.2 ± 23.8 min, p<0.001), greater blood loss (656.8 ± 155.6 vs. 319.6 ± 127.9 mL, p<0.001), and longer hospital stays. EC demonstrated superior functional outcomes (MSTS: 25.5 ± 3.2 vs. 22.1 ± 3.8, p=0.007). Complication rates were higher in SR (35.3%) compared to EC (20.8%), though not statistically significant (p=0.268).

Conclusion

EC offers superior functional outcomes with a non-significant trend toward higher recurrence, whereas SR provides better local control at the cost of greater surgical morbidity. These findings suggest that patient selection should consider tumor grade, extent, and individual functional priorities. Prospective studies are needed to refine treatment algorithms and optimize outcomes.

## Introduction

Giant cell tumor (GCT) of bone is a benign but locally aggressive neoplasm, accounting for approximately 4-5% of all primary bone tumors and 15-20% of benign bone tumors globally [1]. It predominantly affects young adults between 20 and 40 years of age, with a slight female predominance [2]. The knee joint is the most frequently involved site, with the distal femur and proximal tibia together comprising 50-60% of all GCT cases [3]. These tumors are often associated with osteolytic bone destruction, cortical thinning, and soft tissue extension, which can result in pain, reduced mobility, and pathological fractures [4].

Surgical management remains the cornerstone of treatment for GCTs around the knee. The two most commonly employed approaches are extended curettage (EC) and segmental resection (SR), each with distinct indications, advantages, and limitations. EC involves intralesional tumor removal followed by the application of local adjuvants, either chemical (e.g., phenol-based agents), thermal (e.g., cryotherapy or high-speed burr drilling), or mechanical, to reduce residual tumor cells and lower recurrence rates [5]. This technique aims to preserve the native joint, often resulting in superior functional outcomes, with reported Musculoskeletal Tumor Society (MSTS) scores averaging 85-90% [6]. Nevertheless, recurrence rates remain a significant concern, especially in cases of inadequate curettage, with rates as high as 50% [7].

In contrast, SR entails en bloc removal of the tumor-bearing bone segment and subsequent reconstruction using prostheses, allografts, or autografts. SR is typically favored in cases with extensive cortical destruction, pathological fractures, soft tissue extension, or when adequate margins cannot be achieved with intralesional methods [8]. While SR substantially reduces the risk of recurrence to below 5%, it is often associated with longer operative time, higher surgical morbidity, and decreased functional outcomes, typically reflected in MSTS scores averaging 70-80% [9].

Although several studies have evaluated the outcomes of EC and SR, the optimal surgical strategy remains debated. Existing literature often focuses on either oncological safety or functional recovery, but few studies comprehensively compare both parameters alongside complications in a single cohort. Furthermore, long-term outcomes such as joint stability and quality of life remain underexplored.

Therefore, this retrospective study was undertaken to compare extended curettage and segmental resection in patients with GCTs around the knee, specifically assessing recurrence rates, functional outcomes, and postoperative complications. By integrating these outcomes, the study aims to provide evidence-based guidance for surgical decision-making, especially in cases where preserving joint function must be balanced against oncologic safety.

## Materials and methods

Study design and setting

This retrospective observational study was conducted in accordance with the STROBE (Strengthening the Reporting of Observational Studies in Epidemiology) guidelines to ensure transparency and reproducibility. It was carried out in the Department of Orthopaedics at Indira Gandhi Institute of Medical Sciences, Sheikhpura, Patna, Bihar, India. The study analyzed patient records over a six-year period from June 2015 to May 2021. This timeframe was selected based on institutional data availability and to achieve an adequate sample size representative of trends in surgical management during that period. Ethical approval was obtained from the Institutional Ethics Committee (IEC No: 334/IEC/IGIMS/2021, Dated: 13/12/2021).

Patient selection

Inclusion criteria consisted of patients aged 18 years and above with a biopsy-confirmed diagnosis of giant cell tumor (GCT) localized around the knee joint who had undergone surgical intervention, either extended curettage (EC) or segmental resection (SR). All patients underwent preoperative evaluation with magnetic resonance imaging (MRI) to assess tumor extent and joint involvement. Metastatic disease at presentation was ruled out using thoracoabdominal imaging (CT chest and abdominal ultrasonography) and bone scans where clinically indicated. Patients were excluded if they had metastatic GCT or if the tumor was located outside the knee region (e.g., distal radius, sacrum, spine), as these sites exhibit different anatomical and biomechanical characteristics that could confound comparisons in surgical outcomes and recurrence rates.

Surgical techniques

**Figure 1:**
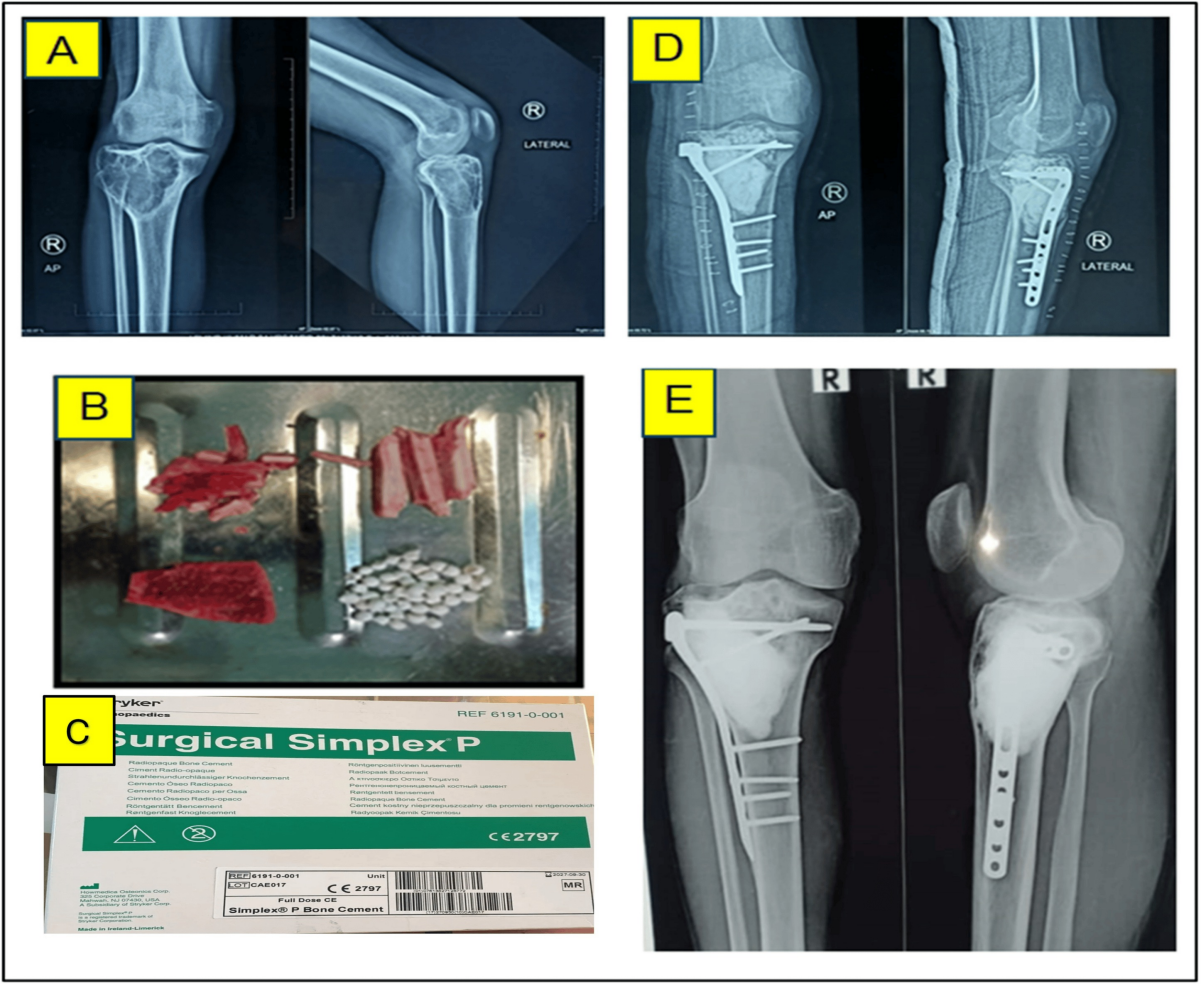
Extended Curettage (EC) in a 32-year-old male. (A) Preoperative X-ray of right proximal tibia showing giant cell tumor (GCT); (B) Intraoperative image depicting iliac crest graft, fibular bone graft, and artificial bone substitute; (C) Bone cement; (D) Postoperative X-ray of the right knee; (E) Four-year follow-up X-ray demonstrating new bone formation at the subarticular surface of the right proximal tibia

Surgeries were performed by a dedicated orthopedic oncology team under standardized protocols to minimize inter-surgeon variability. Patients were divided into two groups based on the surgical procedure received:

Extended curettage (EC) group: Tumor removal was performed through intralesional curettage, followed by adjuvant therapy for recurrence prevention. Adjuvants were categorized as chemical (e.g., phenol, iodine tincture at 10% concentration, and hydrogen peroxide) and mechanical (e.g., high-speed burr drilling and electrocautery). The resulting defect was reconstructed using an autologous iliac crest bone graft combined with artificial bone placed beneath the articular surface. A fibular strut graft (minimum 10 mm thick) was positioned for additional support, and the cavity was subsequently filled with polymethylmethacrylate (PMMA) cement using the sandwich technique. Internal fixation was performed using anatomical plates and screws (Figure 1).

**Figure 2:**
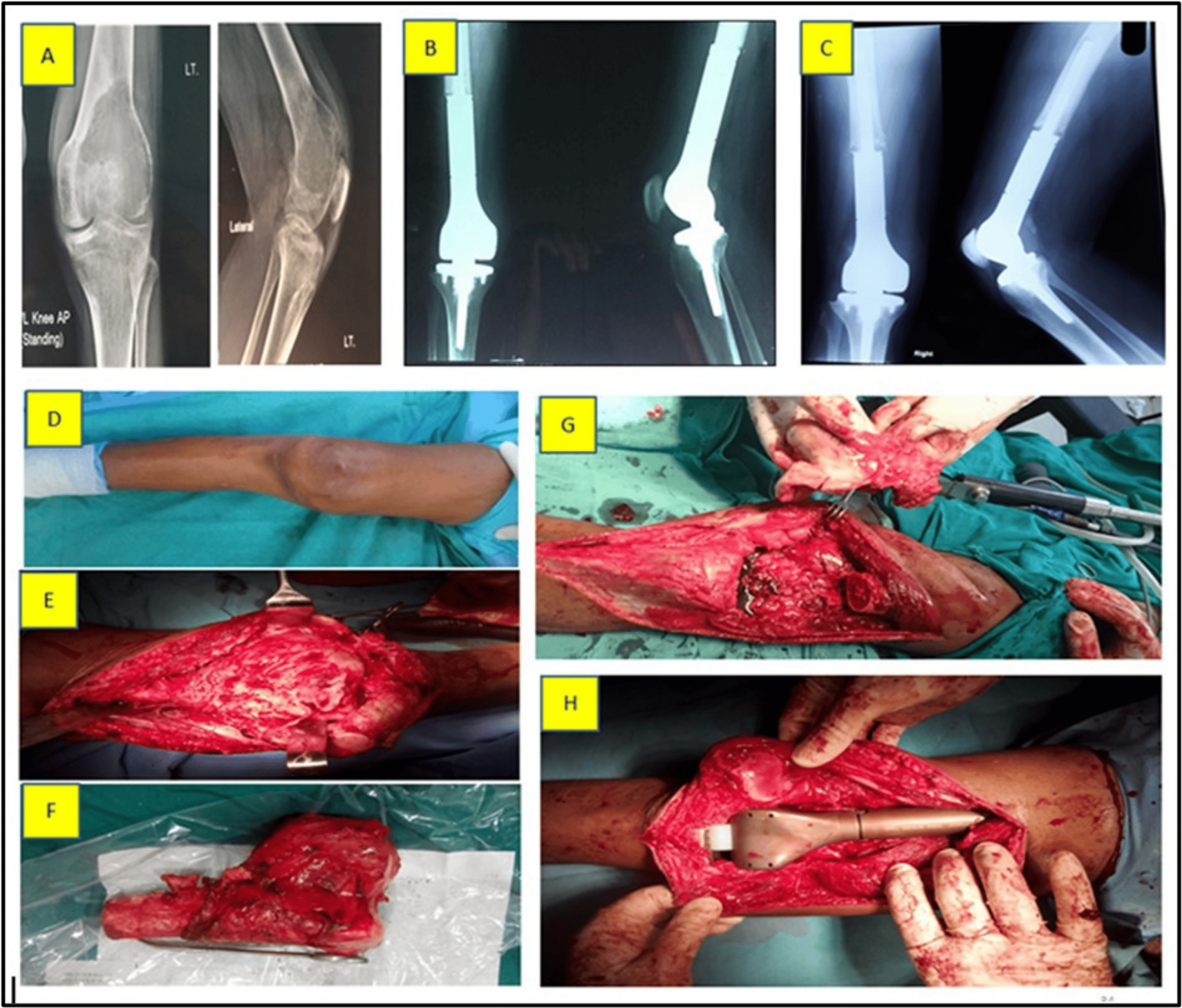
Segmental resection (SR) in a 45-year-old female. (A) Preoperative X-ray of the right distal femur showing giant cell tumor (GCT); (B) X-ray at three months postoperatively; (C) X-ray at three-year follow-up; (D) Preoperative clinical photograph surgical view of the right lower limb; (E) Exposed tumor; (F) Resected distal femur with tumor; (G) Bone gap following resection; (H) Bone gap reconstructed using a hinge knee mega prosthesis

Segmental resection (SR) group: Enbloc resection of the tumor-bearing segment was performed in cases with extensive cortical breach, articular involvement, pathological fracture, or extraosseous soft tissue extension. Reconstruction was done using a custom-modular tumor megaprosthesis. Implant stability was assessed intraoperatively based on fixation integrity, and immediate postoperative imaging (X-ray) was used to confirm component alignment and soft tissue integration (Figure 2).

Postoperative rehabilitation

Rehabilitation protocols were tailored to each group. In the EC group, partial weight-bearing was initiated upon radiological evidence of graft and cement integration. In contrast, patients in the SR group commenced immediate weight-bearing depending on intraoperative assessment of implant stability and postoperative wound healing.

Data collection

Clinical and demographic data (age, sex, tumor location, symptoms), imaging findings (Campanacci grade, MRI features), and surgical details (type of procedure, adjuvants used, operative duration) were extracted from hospital records. Follow-up data included recurrence (clinical or radiological evidence), complications (e.g., infection, joint stiffness, prosthetic loosening, deep vein thrombosis), and functional outcomes assessed using the Musculoskeletal Tumor Society (MSTS) scoring system. This system evaluates six domains: pain, function, emotional acceptance, support requirement, walking ability, and gait, with each domain rated on a scale from 0 to 5 (maximum score of 30) [10]. The MSTS score was chosen due to its validation in musculoskeletal oncology and its sensitivity in assessing limb-salvage outcomes.

Follow-up protocol

Patients were followed up at three-month intervals for the first year, then at six-month intervals for two additional years, and annually thereafter. Local recurrence was monitored over a minimum period of 24 months in all patients using serial radiographs and MRIs where necessary.

Outcome measures

The primary outcome was the rate of local recurrence, defined by clinical signs and radiological or histopathological confirmation of tumor regrowth. Secondary outcomes included functional outcomes (MSTS scores) and postoperative complications. All outcomes were compared between the EC and SR groups to evaluate the oncological safety and functional viability of each approach.

Statistical analysis

All analyses were performed using IBM Corp. Released 2011. IBM SPSS Statistics for Windows, Version 20.0. Armonk, NY: IBM Corp. Descriptive statistics summarized patient characteristics. Categorical variables were analyzed using the chi-square or Fisher’s exact test. Continuous variables were assessed for normality using the Shapiro-Wilk test; parametric data were compared using independent t-tests, while non-parametric data were analyzed using the Mann-Whitney U test. Kaplan-Meier survival analysis was used to estimate recurrence-free survival, with log-rank tests applied for group comparisons. A p-value of < 0.05 was considered statistically significant.

## Results

A total of 65 patients with giant cell tumors (GCTs) around the knee were included, with 48 in the extended curettage (EC) group and 17 in the segmental resection (SR) group. The mean age was similar between the groups (p = 0.427), with a slight male predominance in both. Tumors were predominantly located in the distal femur (p = 0.858). The proportion of Campanacci Grade III tumors was significantly higher in the SR group (70.6%) compared to the EC group (37.5%) (p = 0.031). Pathological fractures were more common in the SR group (41.2% vs. 20.8%), although the difference was not statistically significant (p = 0.121, chi-square test). Preoperative MSTS scores were significantly lower in the SR group (p = 0.008, independent t-test), reflecting poorer baseline function. Follow-up duration was comparable between the groups (p = 0.306) (Table 1).

**Table 1:** Demographic and tumor characteristics of patients undergoing extended curettage and segmental resection *p<0.05 indicates statistical significance. Statistical tests used: t-test for continuous variables and chi-square test for categorical variables MSTS: Musculoskeletal Tumor Society

Variable	Extended Curettage (n=48)	Segmental Resection (n=17)	Test Statistic	p-value
	Frequency (%)/mean ± SD			
Age (years)	31.5 ± 8.2	33.8 ± 7.5	t = 0.801	0.427
Gender				
Male	27 (56.3%)	10 (58.8%)	χ² = 0.019	0.893
Female	21 (43.7%)	7 (41.2%)		
Tumor Location				
Distal Femur	30 (62.5%)	11 (64.7%)	χ² = 0.031	0.858
Proximal Tibia	18 (37.5%)	6 (35.3%)		
Campanacci Grade				
Grade II	30 (62.5%)	5 (29.4%)	χ² = 4.637	0.031*
Grade III	18 (37.5%)	12 (70.6%)		
Pathological Fracture	10 (20.8%)	7 (41.2%)	χ² = 2.422	0.121
Preoperative MSTS Score	23.5 ± 2.8	21.8 ± 3.2	t = 2.746	0.008
Follow-up Duration (months)	38.7 ± 12.4	42.1 ± 10.6	t = 1.034	0.306

The SR group had significantly longer operative time (172.7 ± 36.3 vs. 119.2 ± 23.8 minutes, p < 0.001) and higher intraoperative blood loss (656.8 ± 155.6 vs. 319.6 ± 127.9 mL, p < 0.001), as determined by independent t-tests. All EC cases involved bone grafting and cementing due to post-curettage defects. Hospital stay was significantly longer in the SR group (9.5 ± 2.3 vs. 4.2 ± 1.6 days, p < 0.001), while time to full weight-bearing was significantly shorter (2.5 ± 0.8 vs. 8.7 ± 2.1 weeks, p < 0.001) (Table 2).

**Table 2:** Intraoperative and postoperative surgical parameters in both treatment groups *p<0.05 indicates statistical significance. Statistical tests used: t-test for continuous variables

Variable	Extended Curettage (n=48)	Segmental Resection (n=17)	Test Statistic	p-value
	Frequency (%)/mean ± SD			
Surgical Duration (minutes)	119.2 ± 23.8	172.7 ± 36.3	t = 6.253	<0.001*
Blood Loss (mL)	319.6 ± 127.9	656.8 ± 155.6	t = 8.906	<0.001*
Use of Bone Graft	48 (100.0%)	0 (0.0%)	_	_
Use of Bone Cement	48 (100.0%)	17 (100.0%)	_	_
Hospital Stay (days)	4.2 ± 1.6	9.5 ± 2.3	t = 9.825	<0.001*
Time to Full Weight-Bearing (weeks)	8.7 ± 2.1	2.5 ± 0.8	t = 13.263	<0.001*

Postoperative MSTS scores demonstrated significantly better results in the EC group for pain (p = 0.041), function (p = 0.033), support needed (p = 0.017), and walking ability (p = 0.029). Emotional acceptance was similar between groups (p = 0.125). The mean total MSTS score was higher in the EC group (25.5 ± 3.2) compared to the SR group (22.1 ± 3.8) (p = 0.007), indicating better overall function. The improved function in EC may be attributed to the preservation of native joint structures, particularly contributing to superior walking ability and pain scores (Table 3).

**Table 3:** Functional outcomes based on MSTS scores in extended curettage and segmental resection *p<0.05 indicates statistical significance. Statistical test used: t-test for all variables MSTS: Musculoskeletal Tumor Society

MSTS Domains	Extended Curettage (n=48)	Segmental Resection (n=17)	Test Statistic	p-value
	mean ± SD			
Pain	4.2 ± 0.6	3.8 ± 0.7	t = 2.091	0.041*
Function	4.1 ± 0.7	3.5 ± 0.8	t = 2.203	0.033*
Emotional Acceptance	4.3 ± 0.5	4.0 ± 0.6	t = 1.563	0.125
Support Needed	4.0 ± 0.8	3.2 ± 1.0	t = 2.457	0.017*
Walking Ability	4.4 ± 0.5	3.6 ± 0.9	t = 2.255	0.029*
Overall MSTS Score	25.5 ± 3.2	22.1 ± 3.8	t = 2.781	0.007*

The overall complication rate was higher in the SR group (35.3%) compared to the EC group (20.8%), though the difference was not statistically significant (p = 0.268, chi-square test). Surgical site infections were more frequent in the SR group (11.8%) than in the EC group (6.2%) (p = 0.592). Joint stiffness was also more common in SR (29.4% vs. 12.5%, p = 0.149); though not statistically significant, this finding may be clinically relevant due to the extensive soft-tissue dissection in SR. Prosthetic loosening (11.8%) and implant failure (5.9%) were seen exclusively in the SR group, consistent with known complications of mega prostheses, likely due to mechanical stress and host response over time. No cases of deep vein thrombosis were reported in either group (Table 4).

**Table 4:** Postoperative complications in patients undergoing extended curettage and segmental resection *p<0.05 indicates statistical significance. Statistical test used: Chi-square test for categorical variables

Complication	Extended Curettage (n=48)	Segmental Resection (n=17)		Test Statistic	p-value
	Frequency (%)				
Surgical Site Infection	3 (6.2%)		2 (11.8%)	χ² = 0.287	0.592
Prosthetic Loosening	0 (0.0%)		2 (11.8%)	_	_
Deep Vein Thrombosis	0 (0.0%)		0 (0.0%)	_	_
Joint Stiffness	6 (12.5%)		5 (29.4%)	χ² = 2.080	0.149
Implant Failure	0 (0.0%)		1 (5.9%)	_	_
Overall Complication Rate	10 (20.8%)		6 (35.3%)	χ² = 1.230	0.268

**Figure 3:**
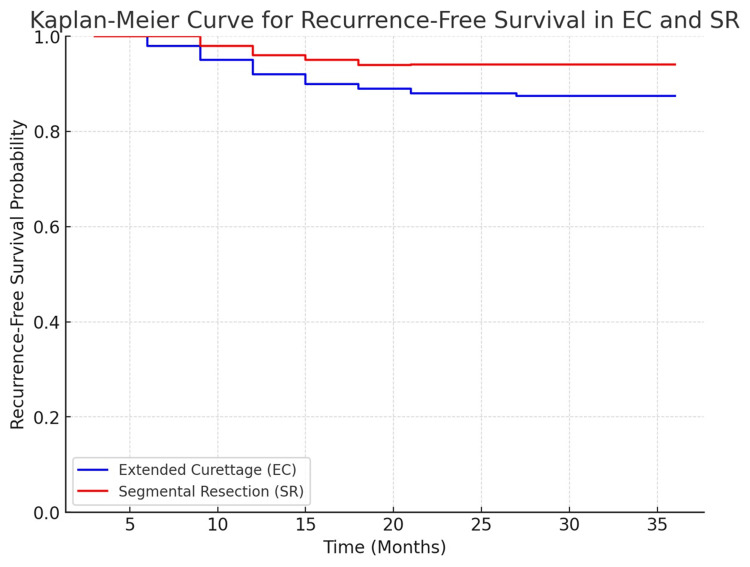
Kaplan-Meier curve for recurrence-free survival in extended curettage and segmental resection

**Figure 4:**
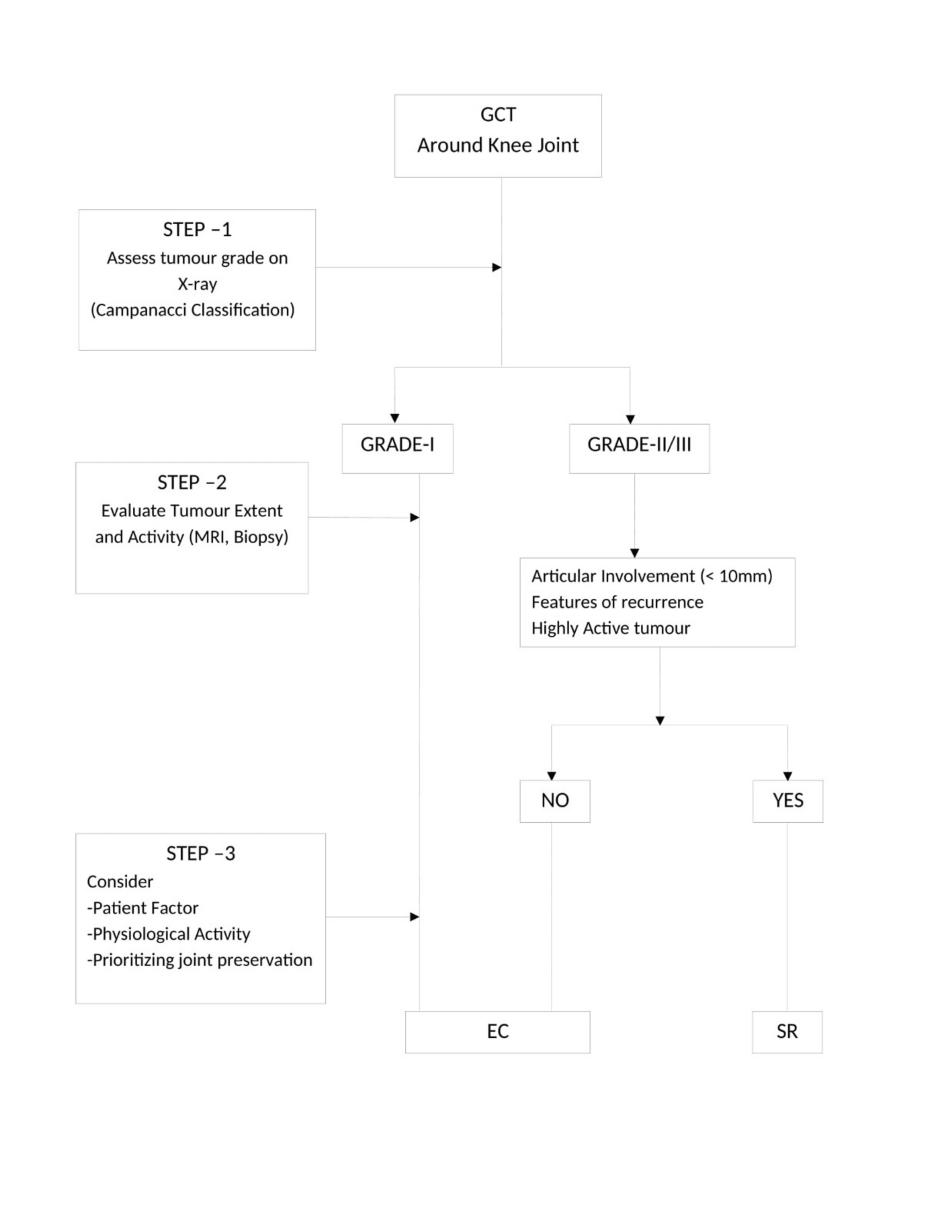
Clinical decision-making flowchart for surgical management of giant cell tumors around the knee

Kaplan-Meier survival analysis revealed similar recurrence-free survival between EC and SR (log-rank test, p = 0.604). At three years, recurrence-free survival was 87.5% in the EC group and 94.1% in the SR group, local recurrence occurred in 12.5% of EC cases and 5.9% of SR cases (p = 0.762, chi-square test). Stratification by Campanacci grade showed recurrence rates of 4.2% (EC) and 0% (SR) for Grade II tumors (p = 0.658) and 8.3% (EC) and 5.9% (SR) for Grade III tumors (p = 0.553). Mean time to recurrence was slightly longer in the SR group (18.2 ± 5.5 months) versus the EC group (14.5 ± 6.8 months) (p = 0.048), indicating a delay in recurrence onset following resection (Table 5 and Figure 3). Importantly, although SR appeared to have a lower recurrence rate, the lack of statistical significance combined with the higher proportion of Campanacci Grade III lesions in the SR group introduces potential confounding. No stratified or adjusted analyses were performed to control for this imbalance, which should be considered when interpreting the comparative oncological efficacy of EC versus SR. Figure 4 shows the clinical decision-making flowchart for the surgical management of giant cell tumors around the knee.

**Table 5:** Recurrence and survival analysis in extended curettage and segmental resection *p<0.05 indicates statistical significance. Statistical tests used: Chi-square test for categorical variables and independent t-test for continuous variables. The log-rank test was used to compare survival distributions.

Variable	Extended Curettage (n=48)	Segmental Resection (n=17)	Test Statistic	p-value
	Frequency (%)/mean ± SD			
Local Recurrence				
Overall	6 (12.5%)	1 (5.9%)	χ² = 0.093	0.762
Grade II	2 (4.2%)	0 (0.0%)	χ² = 0.198	0.658
Grade III	4 (8.3%)	1 (5.9%)	χ² = 0.353	0.553
Mean Time to Recurrence (months)	14.5 ± 6.8	18.2 ± 5.5	t = -2.015	0.048
Recurrence-Free Survival at 3 years (%)	87.5%	94.1%	-	0.604

## Discussion

Giant cell tumors (GCTs) of bone around the knee present unique surgical challenges due to their locally aggressive nature and propensity for recurrence. Our study compared two widely adopted interventions, extended curettage (EC) with adjuvants and segmental resection (SR) with reconstruction, highlighting the trade-off between functional preservation and oncological control.

Local recurrence was observed in 12.5% of EC cases and 5.9% of SR cases, aligning with findings from He et al. and Tsukamoto et al., who reported recurrence rates of <10% for SR versus up to 50% for EC [11,12]. Despite adjuvants like phenol and bone cement, the intralesional nature of EC may leave microscopic tumor cells, contributing to recurrence risk. In contrast, SR ensures en bloc resection, explaining its superior control. Sumari et al.'s meta-analysis reported recurrence rates of 3-8% in SR versus 20-30% in EC, further supporting this oncological benefit [13].

The mean time to recurrence was shorter in EC (14.5 months) than in SR (18.2 months), consistent with Zhang et al., who noted early recurrence (within 18 months) in most post-curettage relapses [14]. This underscores the need for vigilant surveillance, particularly in the first two years after EC.

Three-year recurrence-free survival (RFS) was higher in SR (94.1%) versus EC (87.5%), although this difference was not statistically significant (p=0.604). This trend mirrors prior studies with long-term follow-up [15,16], reinforcing SR's oncological robustness. However, functional compromise remains a critical concern with SR.

Functional outcomes, evaluated via MSTS scores, were significantly better in the EC group (25.5 ± 3.2) versus SR (22.1 ± 3.8, p=0.007). Notably, domains like pain, function, support needed, and walking ability showed marked superiority. This may be attributed to preserved joint biomechanics, minimal disruption of neuromuscular pathways, and reduced soft tissue handling in EC. Studies by Wang et al. and Behera et al. reported similar findings, with EC patients achieving MSTS scores above 85% and better mobility [17,18].

Interestingly, the EC group had delayed full weight-bearing (8.7 vs. 2.5 weeks) due to the need for bone consolidation. Despite this delay, EC patients achieved superior long-term function. SR patients benefitted from immediate prosthetic stability but lacked proprioception, joint articulation, and adaptability, contributing to lower MSTS scores and reduced satisfaction in high-demand activities [19].

SR was associated with significantly longer operative times (172.7 vs. 119.2 minutes) and greater intraoperative blood loss (656.8 vs. 319.6 mL), corroborating Liu et al.'s findings [20]. Hospital stay was also prolonged in SR (9.5 vs. 4.2 days), reflecting the higher surgical burden and postoperative morbidity [21]. To mitigate these risks, advanced surgical techniques, blood conservation strategies, and enhanced perioperative protocols may be implemented, potentially reducing complications and recovery time.

Complications were more frequent in the SR group (35.3% vs. 20.8%), though not statistically significant. Prosthetic loosening and implant failure occurred exclusively in SR, a known long-term risk with megaprostheses. These findings align with Errani et al., who documented prosthetic failure rates of 10-15% within five years [22]. Joint stiffness was also higher in SR (29.4% vs. 12.5%, p=0.149), and although statistically non-significant, the clinical impact is noteworthy. This warrants caution, especially in younger patients with high activity demands.

Surgical site infections were marginally more common in SR (11.8% vs. 6.2%), likely due to longer surgical exposure, foreign material, and higher intraoperative time, consistent with findings by Medellin et al. [23].

Clinical implications

Treatment selection must be individualized, balancing oncological safety with functional demands [24,25]. Segmental resection is preferable in Campanacci Grade III tumors, pathological fractures, and cases with soft tissue involvement or extensive cortical breach [26,27]. Conversely, EC is suited for Grade II lesions with contained involvement, particularly in younger patients prioritizing joint preservation and mobility [28,29]. EC can be done in grade 3 tumors, even if there are extra-compartmental with good articular margin. A visual decision-making algorithm is provided (Figure 4) to guide clinicians in choosing between EC and SR based on tumor characteristics and patient factors.

Limitations

This retrospective study is prone to selection bias, as treatment allocation was influenced by tumor size, grade, and surgeon preference. The higher proportion of Grade III tumors in the SR group may have affected outcomes and confounded recurrence comparisons. Sample size constraints may have limited statistical power, particularly for complication rates and survival analysis. Variability in adjuvant use, despite protocol guidance, introduces further heterogeneity that may impact recurrence rates. Moreover, while MSTS is a validated score, it does not capture subjective aspects like emotional well-being, return to occupation or patient satisfaction. Future studies should incorporate patient-reported outcome measures (PROMs) and consider stratified or adjusted analyses to account for baseline tumor severity.

## Conclusions

This study underscores the distinct clinical profiles of extended curettage (EC) and segmental resection (SR) in the treatment of giant cell tumors (GCTs) around the knee. While EC is associated with significantly superior functional outcomes, as reflected by higher MSTS scores, it carries a comparatively higher risk of local recurrence. Conversely, SR offers better recurrence-free survival and local tumor control but is accompanied by greater surgical complexity, increased blood loss, and longer postoperative rehabilitation. These findings highlight the importance of individualized treatment planning based on tumor characteristics, patient age, functional expectations, and tolerance for recurrence risk. For surgeons, this study provides practical insights into balancing oncologic safety with functional preservation. Further prospective studies with larger cohorts are warranted to explore long-term prosthetic complications, refine the role of adjuvant therapies, and integrate patient-reported outcome measures for a comprehensive understanding of treatment efficacy.
